# Impact of Multifaceted Workplace Bullying on the Relationships between Technology Usage, Organisational Climate and Employee Physical and Emotional Health

**DOI:** 10.3390/ijerph18063207

**Published:** 2021-03-19

**Authors:** Mehwish Iftikhar, Muhammad Imran Qureshi, Shazia Qayyum, Iram Fatima, Sriyanto Sriyanto, Yasinta Indrianti, Aqeel Khan, Leo-Paul Dana

**Affiliations:** 1 NUST Business School, National University of Science and Technology, Islamabad 44000, Pakistan; Mevy.iftikhar@yahoo.com; 2 Faculty of Technology Management and Technopreneurship, Universiti Teknikal Malaysia Melaka, Hang Tuah Jaya, Melaka 76100, Malaysia; 3 Institute of Applied Psychology, University of the Punjab, Lahore 54782, Pakistan; shazia_agha@hotmail.com (S.Q.); iramraheel70@gmail.com (I.F.); 4 Social Studies Department, Universitas Muhammadiyah Purwokerto, Purwokerto 53182, Indonesia; sriyanto1907@gmail.com; 5 Entrepreneurship Department, Podomoro University, Jakarta 11470, Indonesia; yasintaindrianti@gmail.com; 6 Faculty of Social Sciences and Humanities, Universiti Teknologi Malaysia (UTM), Skudai, Johor Bahru 81310, Malaysia; aqeel@utm.my; 7 Rowe School of Business, Dalhousie University, Halifax, NS B3H 4R2, Canada; lp762359@dal.ca

**Keywords:** organisational climate, technology usage, workplace bullying, cyberbullying, emotional intelligence

## Abstract

This research article investigates the effect of organisational climate and technology usage on employees’ physiological and emotional health damage resulting from face-to-face bullying and cyberbullying at the workplace. Furthermore, we investigated emotional intelligence as a coping strategy to moderate employee physiological and emotional health damage. The research used a quantitative research design. A five-point Likert-scale questionnaire was used to collect data from a multistage sample of 500 officials from Pakistan’s four service sectors. Results revealed that organisational climate and technology usage are negatively related to face-to-face bullying and cyberbullying at the workplace. At the same time, workplace bullying adversely affects an employee’s emotional and physiological health. However, emotional intelligence can reduce an employee’s emotional health damage due to workplace bullying. Thus, we suggest incorporating emotional intelligence training at the workplace to minimise the devastating effects of face-to-face bullying and cyberbullying on employees’ physical and emotional health.

## 1. Introduction

Today’s contemporary organisations have a particular focus on workplace bullying. Workplace bullying attributed to systematic power abuse may be adopted in various forms, including predatory, job-related, and individual-related bullying; direct or indirect harassment; and dispute-related bullying, either face-to-face or online known as cyberbullying [1,2]. Workplace bullying (WB) has been described by Salin [3] as ‘repeated and persistent negative acts towards one or more individual(s), which involve a perceived power imbalance and create a hostile work environment’. The prevalence of workplace bullying varies considerably. Based on country, industrial sector and method of measurement [4,5], bullying rates of 25% in Portugal [6], 13.6% in the Czech Republic [7], 16.2% in the telecom sector of Canada [8] and 30% face-to-face bullying victimisation among adults [9] have been reported. Both face-to-face bullying and cyberbullying were detected in the educational settings in Spain [10].

It was revealed by Privitera and Campbell [11] that cyberbullying is a new form of harassment that has emerged with technological advancement. Still, practices to use these resources have not evolved simultaneously. Cyberbullying is defined as ‘inappropriate, unwanted social exchange behaviours initiated by a perpetrator via online or wireless communication technology and devices’ [11]. It also indicates the modern world has fewer boundaries when it comes to personal contact, even in the sphere of harassment [12]. Zhao et al. [13] depicted that cyberbullies can reach their targets in any location, at any time, through different means, like phone, e-mail, social networking sites and text messaging. With the excessive use of technology, bullies are inevitable, so technology usage is essential for work and family communication [14,15]. Workplace bullying involves frequent contact by peers, managers, or direct accounts over a period of at least six months to carry out acts of maltreatment and abuse and show aggressive behaviour [1,16].

Most organisations are deploying and promoting technology at workplaces. The organisational climate has been altered with excessive technology use that allows workers to use social media at the workplace. The role of Information and Communication Technologies (ICT) is significant in the personal development of individuals and organisations and boosts the economy. Besides its benefits, it also reshapes the behaviours of employees at the workplace, which directly affects the traditional norms of the organisation. On the other hand, it is becoming a challenging job for organisations to continuously update and revise their rules and regulations to overcome cyberbullying in a dynamic technological environment.

Workplace bullying has become a global issue that needs urgent attention, as it may have harmful effects on workers’ emotional, psychological and physical health. Victims of bullying have an increased possibility of mental and psychological health damage, like anxiety, depression, increased alcohol usage, high levels of blood pressure, insomnia and headache [17,18]. Corney [19] stated that workplace-bullying victims are more inclined towards suicidal attempts in extreme cases.

Braun [20] indicated that nearly 30% of the participants surveyed had experienced face-to-face harassment at work at some point in their careers. A survey showed that 27% of workers faced harassment [21], and 30% reported face-to-face bullying among middle-aged employees at work [9]. At the same time, somewhere between 9% and 20% of workers from various occupations reported victimisation through cyberbullying at work [11,22,23]. As technology is playing an increasingly significant role at work, because of its improved abilities, harassment and specifically cyberbullying are becoming a more pressing problem [12]. Modern organisations are consistently witnessing behaviour adaptations due to ever-increasing access to digital technologies. Thus, it has become quite challenging for organisations to come up with measures to tackle this situation effectively and mitigate workplace bullying.

Workplace bullying, either face-to-face bullying or cyberbullying, is becoming a global issue [24] and resulting in physical and psychological health issues for employees in organisations [25]. The organisational climate tends to make its employees vulnerable to face-to-face bullying and even cyberbullying. However, the evidence claiming that organisational climate and technology usage at the workplace can damage an employee’s physical and emotional health through both face-to-face bullying and cyberbullying is not sufficient. For this reason, we aimed to investigate the mediating effect of face-to-face bullying and cyberbullying on the relationship of organisational climate and technology usage with an employee’s physical and emotional health damage.

The second fundamental question is, how do you reduce workplace bullying? Organisations must take care of the emotional health damage among their employees because this will reduce the job burnout ratio. Further, emotional intelligence helps understand workers’ and employees’ sensitivity and reduces health damage. Emotional intelligence is a set of skills that can act as an affinity within the working environment [24]. Thus, we aimed to investigate the moderating effect of emotional intelligence on the relationship between workplace bullying and an employee’s physical and emotional health damage.

## 2. Literature Review

### 2.1. Theoretical Development

The theoretical development of workplace bullying and employee health needs to be considered in terms of different aspects in an ICT context. For example, first we need to understand the person–environment fit. Most of the researchers in their studies have proposed various models that come up with many other concepts together that summarise the interaction of the working environment and the individual characteristics presented through a framework [26]. Thus, the person–environment fit highlights the importance of an individual’s interaction with a particular working environment provided by the employer. According to Spector and Fox’s [27] counterproductive workplace behaviour model (CWB model), occupational stress and aggression have a direct influence on an employee’s behavioural responses, cognition and emotions in a working environment. For example, in the context of behavioural response, the worker can easily manage stressful situations and avoid inappropriate confrontations with others. However, it is an alarming or threatening situation for the organisation’s well-being and others involved [28].

The organisational culture and climate is another aspect that affects workplace bullying. Qureshi et al. [29] proposed a three-way model in which they highlighted the role of frustration among employees, the intensity of rising conflicts, and the culture of dividing employees into teams in bullying. This model indicates that bullying is created due to the organisation’s inability to manage frustration among employees. Felson and Tedeschi [30] confirmed the same in revised frustration aggression theory (RFAT) and social interactionist theory (SIT), respectively. Thus, the bullying behaviour of employees directly originates from the culture and climate of the organisation. Leymann [31] addressed the working environment and its influence on bullying. The proposed hypothesis is that ’disappointment and stress among employees may lead to bullying due to the management’s negligence in a negative psychosocial environment’.

Based on the system thinking, Johnson [32] also developed an ecological model that defined the working environment as a series of hierarchical structures (interconnected) that exist in our society. These interconnected layers are the corporation (exosystem), the society (macrosystem), the target and the bully (microsystem) and the managers and co-workers (mesosystem). Johnson [32] said that workplace bullying is not created due to a state of isolation; rather, it is preceded by each of these layers, and identifying effective responses to and informing all about these interconnected opportunities are essential. Similarly, Oliveira [33] indicated that contemporary organisations’ technology is a driving force that shapes individual behaviour. Therefore, technology has a direct influence on reshaping human and individual behaviour.

### 2.2. Organisational Climate and Technology Usage as a Precursor of Workplace Bullying

Bullying acts as a psychosocial hazard at work and is associated with an organisation’s structure and culture [34]. It is also related to some extent to the behaviours of the leaders at the workplace. The sources of victimisation have been identified as conflict, incompetence to resolve that conflict and socially inept exposure of individuals in the environment of the organisation [35]. ‘Insufficient staff, i.e., inadequate relational care, role conflicts, poor leadership, and lack of decision-making autonomy’ are also risk factors for organisations [36]. It is vital to understand how human resource managers can help create a safe working situation free from victimisation, thus giving workers a chance to have a relaxed working environment free from psychological, physical and emotional stress so that workers remain healthy physically, emotionally and psychologically. Managers in organisations need to take appropriate actions to monitor, evaluate and lessen this severe issue’s adverse outcomes. Accordingly, we formed the following hypotheses:

**Hypothesis** **1.** *The organisational climate has a negative relationship with face-to-face bullying.*

**Hypothesis** **2.** *The organisational climate has a negative relationship with cyberbullying.*

The rapid use of technology increases the danger of being involved in the alternative way of bullying, i.e., cyberbullying, facilitated by technology. Lawrence [23] argued that cyberbullying is distinctive from face-to-face victimisation and is found to be more harmful. Cyberbullying involves ‘fraudulent, aggressive, anonymous, hacking into e-mail accounts, unwanted messages, threats, spreading rumours, harassment, unwanted phone calls, malicious, abusive messages’. The essential element for cyberbullying is the use of technology. Organisations where ICT is excessively used tend to be prone to face-to-face bullying and cyberbullying. Thus, we formed the following hypotheses:

**Hypothesis** **3.** *Technology usage at the workplace has a negative relationship with face-to-face bullying.*

**Hypothesis** **4.** *Technology usage at the workplace has a negative relationship with cyberbullying.*

### 2.3. Associations between Face-to-Face Bullying, Cyberbullying and Employee Health

Einarsen [37] and Savicki, Cooley and Gjesvold [38] conducted multiple studies and concluded that bullying has a robust correlation with psychosomatic health and psychological well-being; if not addressed, it can result in the experience of burnout in terms of emotional health damage. Another study conducted in hospitals and some other organisations by Kivimäki, Elovainio and Vahtera [39] showed that workplace bullying is related to self-reported fatigue and is associated with the intension to quit the job. A study conducted by Einarsen [37] on 745 Norwegian medical staff showed that bullied workers experience a higher level of exhaustion compared to non-bullied colleagues. Mathisen, Einarsen and Mykletun [40] conducted another Norwegian study and explored the incidence and effects of harassment in restaurants. Results of the study demonstrated a definite link between experience of victimisation behaviour and burnout.

Past research has established that workplace bullying, as a considerable stressor, negatively affects the affected victims’ well-being and has adversative effects [41]. Parkins et al. [42] showed that workplace bullying might have severe outcomes in terms of mental health and physical well-being. Emotional and physical symptoms consist of despair, jittery feelings, lack of ability to think, nervousness, petulant feelings, annoyance, digestion problems, high blood pressure, depression, sleep disturbance, etc. The discussion leads to the following hypotheses:

**Hypothesis** **5.** *Face-to-face bullying has a positive effect on an employee’s emotional health damage.*

**Hypothesis** **6.** *Face-to-face bullying has a positive effect on an employee’s physical health damage.*

Limited research has documented the impact of cyberbullying at work. Baruch’s [22] study depicted that harassment via e-mail is allied with turnover intentions in organisations, lower job satisfaction levels, and anxiety and depression. Lawrence [23] revealed that online bullying has more severe and substantial effects than off-line bullying. Okoiye et al. [43] depicted in their study that cyberbullying consists of harassment by the offender against a physically distant victim. Though in cyberbullying, the offender and the victim do not have personal contact, it is still emotionally and psychologically destructive for the victim. This sort of destruction might produce strain that provokes the victim towards negative behavioural selection and stimulates feelings of dissatisfaction, anger and despair. Negative behavioural selection refers to the process by which individuals have more frequent mood and behaviour changes, most of the time provoking sadness, anxiety and anger. Thus, we hypothesised the following:

**Hypothesis** **7.** *Cyberbullying has a positive effect on an employee’s emotional health damage.*

**Hypothesis** **8.** *Cyberbullying has a positive effect on an employee’s physical health damage.*

### 2.4. Emotional Intelligence as a Coping Strategy

Burnout is a concern of health organisations and a syndrome that might affect every organiation’s workers. Several factors may contribute to reducing or preventing the level of burnout among workers. Tsaousis and Nikolaou [44] underline that organisations must endeavour to avoid burnout among their workers, and emotional intelligence seems to reduce the possibility of burnout. Burnout is somewhat similar to depression [45], and one of the factors of emotional intelligence, i.e., emotion management, might reduce fatigue. Increased levels of exhaustion are also related to decreased levels of compassion [46]. Emotional intelligence is a skill that can depreciate [47] and can affect the level of empathy based on the work environment. Emotional intelligence must be fostered in organisations to maintain worker empathy and reduce burnout [47].

Tsaousis and Nikolaou [44] proposed that increased emotional intelligence might improve physical and mental health related to stress-reducing behaviours. Kaur [48] proved a positive and significant impact of emotional intelligence on mental health. Moreover, emotional coping might help reduce occupational stress [47]. An emotionally intelligent person may have control over his/her emotions, which induces behaviours that may help in stress reduction [49], conflict management and ethical concerns [50]. Furthermore, individuals can identify their emotions based on emotional intelligence (EI) and can considerately reflect on the effect of these emotions [47,51,52,53]. In other findings, EI was proved to be a variable that may alleviate work and organisational stress [54]. EI also helps individuals get a better insight into emotions and the consequent reactions associated with stressful stimuli, which ultimately lessens stress and emotional health damage like burnout [55].

Therefore, in this research, emotional intelligence is proposed as a handling strategy to overcome the negative health impacts of workplace bullying. Emotional intelligence can be vital to overcome negative health impacts. However, limited research has been conducted to uncover emotional intelligence’s effects on the link between workplace bullying and employee health damage. We hypothesised that the following:

**Hypothesis** **9.** *Emotional intelligence moderates the relationship between face-to-face bullying and emotional health damage.*

**Hypothesis** **10.** *Emotional intelligence moderates the relationship between cyberbullying and emotional health damage.*

**Hypothesis** **11.** *Emotional intelligence moderates the relationship between face-to-face bullying and physical health damage.*

**Hypothesis** **12.** *Emotional intelligence moderates the relationship between cyberbullying and physical health damage.*

**Figure 1:**
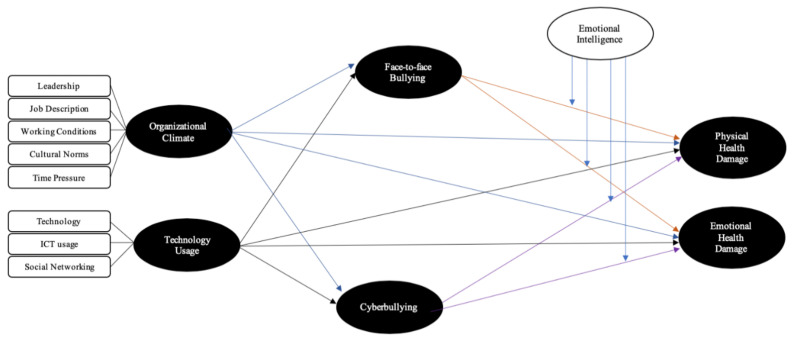
Conceptual framework (self-developed).

The conceptual framework of the study was developed as presented in Figure 1.

## 3. Methodology

### 3.1. Measures

Measures were adapted from previous studies (standardised questionnaires) and amended according to the current study’s requirements. The Majer D’Amato Organizational Questionnaire 10 (MDOQ10) [56] was used to measure organisational climate, the Negative Acts Questionnaire-Revised (NAQ-R) by Giorgi [57] was used for face-to-face bullying and the Cyberbullying Scale (CBS) by Çetin, Yaman and Peker [58] was used for cyberbullying.

The General Health Questionnaire (GHQ) is a well-known measure of current mental health since Goldberg’s development [59] in the 1970s. It has been used in a variety of organisational settings in various cultures extensively. Initially, the questionnaire was developed as a 60-item instrument, but currently, a range of shortened versions of the questionnaire, like GHQ-30, GHQ-28, GHQ-20 and tGHQ, is available. In this study, the researcher adopted some of the items from the shortest version, GHQ-12 (with a 12-item scale), adapted from the General Health Questionnaire by Goldberg [59].

The Physical Health Questionnaire (PHQ), a self-report scale, was adapted (some items according to the study) to measure physiological health in the current study. The Physical Health Questionnaire (PHQ) is a somewhat brief measure of physiological health. The scale was adapted from the Physical Health Questionnaire (PHQ) by Schat, Kelloway and Desmarais [60].

Emotional health damage was measured based on the Maslach Burnout Inventory Scale [61]. The Wong and Law Emotional Intelligence Scale (WLEIS) [62] was adapted to assess emotional intelligence. The items for technology usage were self-developed and pre-tested accordingly. Technology usage refers to the use of computer and ICT for official purposes and is provided by organisations to their employees to perform their jobs at the workplace.

A 5-point Likert-scale questionnaire was used to collect data. Before conducting the final survey, the questionnaire was pilot-tested to test the instrument’s validity and reliability. Data were collected by two means of communication, that is, online and face-to-face. Schillewaert [63] described that for more general topics, such as consumer goods and lifestyle issues, living habits, attitudes, opinions and interests, off-line and online samples seem to generate results that are not significantly different from one another. Hence, it was not problematic to use both online and off-line data collection methods in the current research; the questions are related to the attitudes and perceptions of workers working in the service sector.

### 3.2. Population and Sample

This study’s target population was the workforce in the service sector, mainly Pakistan’s telecommunications, banking, hoteling and education sectors. A random sampling technique spread over various stages was applied. The sampling framework constituted all public sector banks, telecommunication organisations, hotels with a rating above four stars and educational institutions recognised by the Higher Education Commission, Pakistan. A multistage sampling technique was applied. In the first stage, a random sampling technique was used for the choice of each subsector. In Pakistan, the service sector consists of four natural sectors (distributive, producer, personal and social services), with many subsectors in each. One subsector from each of the four natural sectors was selected randomly.

In the second stage of multistage sampling, disproportionate stratified sampling was used. There are 6 telecommunication companies, 33 banks, 29 hotels and 179 universities situated in various regions in Pakistan. Each subsector/subgroup is non-overlapping, with a different number of companies, hence forming four strata. In this sampling stage, five companies were selected from each stratum based on disproportionate sampling. The sampling fraction to be applied in the telecommunication sector was 1 in 1 approximately, the sampling fraction applied in the banking stratum was around 1 in 7 and the sampling fraction to be used in hotels and education was 1 in 6 and 1 in 35 (approximately), respectively. Hence, disproportionate stratified random sampling allowed five telecommunication companies, five banks, five hotels, and five higher educational institutes to be selected. In the third stage, 500 officials from Head Quarters were selected based on purposive sampling. Workplace bullying was measured based on bullying occurrences during the past six months or more, so the questionnaires were circulated among respondents who had been working in that organisation for more than six months in the main branches. Furthermore, most of the main offices are situated in Pakistan’s capital cities, like Karachi, Lahore and Islamabad, and can be considered true representatives of the population due to the ethnic diversity in the major cities.

## 4. Results

Due to the complexity of the model, we used partial least squares–structural equation modelling (PLS-SEM) using the SmartPLS v. 3.2.8 (*SmartPLS GmbH*, Boenningstedt, Germany) [64] statistical tool to assess the measurement and structural model, as it can adjust smaller sample sizes with no normality assumptions [65]. This study followed Anderson and Gerbing’s [66] guidelines and tested the measurement model using a two-step approach, followed by evaluation of the structural model [66]. The measurement model was assessed to ensure the validity and reliability of the items and constructs. The later stage was to evaluate the structural model to test the hypotheses of the study.

### 4.1. Measurement Model Assessment

To evaluate the measurement items and constructs, we tested both convergent and discriminant validity. The tests for reliability and convergent validity are presented in Table 1. This research employed composite reliability to assess reliability, and values more than 0.7 were considered sufficient [67,68]. Convergent validity evaluates the degree to which items are related to the construct as theoretically conceptualised. Convergent validity was tested using the item loadings and the average variance extracted (AVE) for each construct [67,68]. Table 1 shows the results of the measurement model. All item loadings surpassed 0.7, and the AVE surpassed 0.50 for all constructs, indicating sufficient convergent validity in the measurement model. All items having a factor loading of less than 0.650 were deleted to maintain the AVE value above 0.50. The factor loading values for organisational climate ranged from 0.794 to 0.930, with a composite reliability value of 0.933 and an AVE of 0.736. Factor loading for technology usage at the workplace ranged from 0.889 to 0.930, with a composite reliability value of 0.934 and an AVE of 0.822. Factor loading for face-to-face bullying ranged from 0.663 to 0.862, composite reliability was 0.968 and the AVE was 0.613. The factor loading range for cyberbullying was 0.613 to 0.868, composite reliability was 0.968 and the AVE was 0.668. Similarly, the factor loading ranges for emotional health damage, physical health damage and emotional intelligence were 0.842–0.899, 0.651–0.838 and 0.640–0.820, respectively, with composite reliability values of 0.908, 0.937 and 0.950 and AVE values of 0.767, 0.554 and 0.501, respectively.

Discriminant validity refers to the extent to which an instrument contains a truly distinct construct from all others. Discriminant validity can also be the degree to which similar constructs have distinct values. It implies that a construct is unique and represents its logic in the model. Discriminant validity can be shown when the value of the square root of the average variance extracted should exceed the value of inter-construct correlations. In the current study, two criteria were used to test the discriminant validity of the constructs. These were the Fornell–Larcker criterion and cross-loadings. The Fornell–Larcker criterion refers to the square root of the average variance extracted for each latent construct greater than the latent inter-construct correlation with other latent variables in the model. Table 2 on the Fornell–Larcker criterion shows that the square root of each latent construct’s average variance extracted is greater than the latent inter-construct correlation with other latent variables in the model.

This study tested discriminant validity applying the heterotrait-monotrait ratio of correlations [69], as shown in Table 3. If a heterotrait-monotrait (HTMT) value is greater than 0.85 [70], then there is a discriminant validity problem, whereas if the values are smaller than 0.85, it signals good discriminant validity. As all HTMT values were lower than 0.85 [70], as shown in Table 3, good discriminant validity was ascertained. Both assessments indicate the validity and reliability of measurement items, thus allowing for hypothesis testing.

### 4.2. Structural Model and Evaluation and Hypotheses Testing

As the measurement model or outer models were reliable and valid, the next step was assessing the structural model or the inner model. The process involved examining the model’s predictive capabilities and the relationships between the constructs [71]. In other words, the structural model’s assessment was taken to evaluate the hypothesised relationships within the inner model. Three parameters determine the hypothesised relationships between constructs in the current study, and these criteria are the coefficient of determination (*R*^2^) of endogenous constructs, effect size (ƒ^2^) and path coefficients, and *t*-statistic value. The predictive relevance of the model (Q^2^) and the goodness-of-fit (GOF) index are critical standards for evaluating the inner structural model.

#### 4.2.1. Measuring the Value of R^2^

The structural model quality was assessed through R^2^ values, path coefficients and subsequent *t*-values. This study used a bootstrapping procedure with 5000 resamples [67,68] to obtain a valid *t*-value calculation error. According to Kline [71], the *R*^2^ value ranges from 0 to 1 and the values of 0.75, 0.50 and 0.25 describe substantial, moderate and weak levels of predictive accuracy, respectively. This study first analysed the effect of organisational climate, technology usage and workplace bullying on employees’ physical and emotional health. Organisational climate and technology usage explained 58% of cyberbullying variation and 59% of the face-to-face bullying variation. Organisational climate, technology usage and workplace bullying accounted for 71% variation in emotional health damage and 69% in physical health damage. The values of Variation Inflation Factor (VIF) for all paths were reported as less than 5. These values indicate that there is no issue of multicollinearity in the structural model.

#### 4.2.2. Measuring the Effect Size (f^2^)

The effect size is used to determine whether the omitted construct has a substantive effect on the endogenous constructs. The effect size can be measured by increasing R^2^ relative to the variance of the endogenous latent variable that remains unexplained. As a rule of thumb, Cohen (1988) described that f^2^ values of 0.02–0.14, 0.15–0.34 and greater than 0.35 signify small, moderate and large effects, respectively. Table 4 indicated all f^2^ values obtained in this study were in the range between 0.02 and 0.239; this indicates small and moderate effect sizes.

#### 4.2.3. Predictive Relevance of the Model (Q^2^)

**Figure 2:**
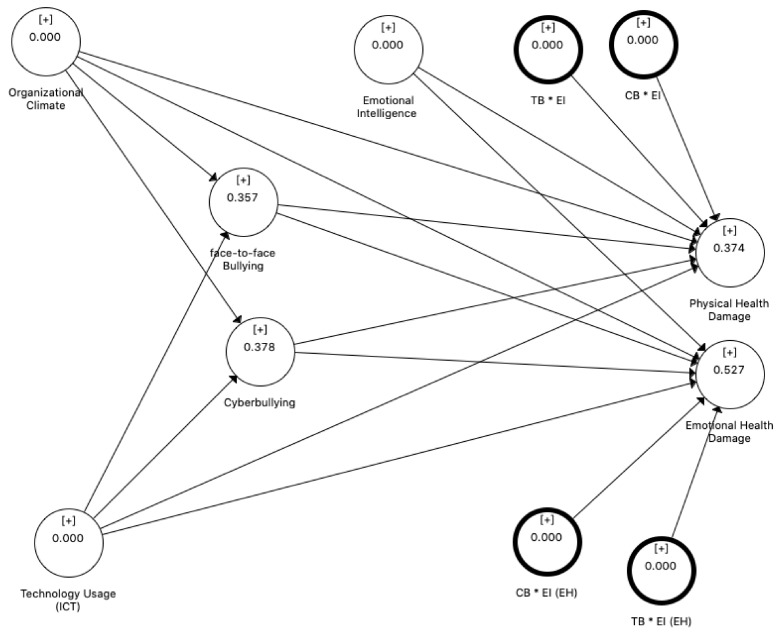
Q^2^ statistics.

The blindfolding procedure was used to calculate the Q^2^ statistics in SmartPLS software. This technique is used to measure the quality of the path model and data fitness. If the value of Q^2^ is greater than zero, it can be considered a conceptual model that can measure the endogenous latent constructs [69]. Figure 2 indicates that the results of Q^2^ statistics revealed that the proposed model can predict the values of endogenous latent constructs. The Q^2^ values of the endogenous constructs face-to-face bullying, cyberbullying, physical health damage and emotional health damage were 0.357, 0.378, 0.374 and 0.527, respectively. These values are higher than the threshold values. Thus, the conceptual model has adequate predictive relevance.

#### 4.2.4. Goodness-of-Fit Index

The goodness-of-fit (GOF) was used to assess the model fitness. The GOF is an index for the complete model fit to verify that the model sufficiently explains the empirical data. The values of the GOF can be between 0 and 1, where GOF > 0.10 but <0.24 indicates a small effect, GOF > 0.25 but <0.35 means a medium effect and GOF > 0.36 indicates a large effect and a global validation of the structural model. ‘A good model fit shows that a model is parsimonious and plausible’ [72]. The goodness-of-fit was calculated with the help of the following equation in this study [72]:

$$GOF\;=\;\sqrt{Average\;R^{2}\times \;Average\;communality}$$

The detailed calculations of the GOF for this study are provided in Table 5. The GOF index value was 0.665 for this study. These values indicate the fitness of empirical data and have substantial predictive power. It means the data fit the proposed model and have substantial predictive power in comparison with baseline values.

#### 4.2.5. The Standardised Root-Mean-Square Residual (SRMR)

Finally, we used the two most frequently used model fit criteria for partial least squares (PLS) path modelling: normed fit index (NFI) and standardised root-mean-square residual (SRMR). The SRMR of the saturated model was 0.043 and for the estimated model was 0.062. Meanwhile, the NFI was reported as 0.901 for the saturated model and 0.892 for the estimated model. Threshold values for SRMR < 0.08 [44] and NFI > 0.90 were recommended as indicators of a good model fit, and the model fit for the study is acceptable, as indicated in Table 6.

#### 4.2.6. Path Coefficients and Hypothesis Testing

**Figure 3:**
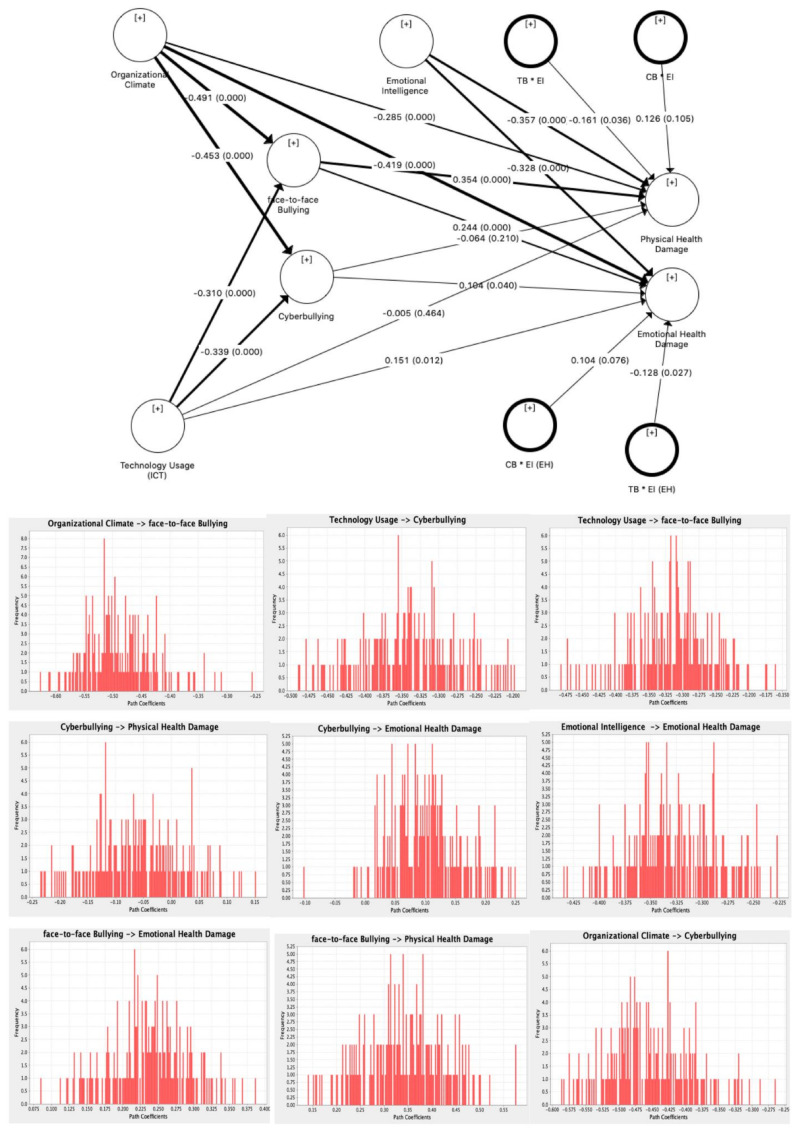
Structural model results.

PLS-SEM uses the path coefficient to determine the hypothesised relationships’ strength and significance between the latent construct. The estimates are obtained for structural model relationships with standardised values between −1.00 and +1.00. A coefficient closer to +1.00 indicates a strong positive relationship, and a coefficient closer to −1.00 shows a strong negative relationship. Figure 2 shows the path coefficients of the model. These path coefficients can also be interpreted as standardised beta coefficients of the OLS. The bootstrapping technique is used to calculate the empirical *t*-value for the path coefficients to test for the significance of hypothesised relationships. The relevance of the significance is thus important as it would warrant managerial attention. Table 7 presents a summary of the hypothesis testing. Figure 3 indicates the bootstrapping results and obtained a normally distributed graph for each path.

The study aimed to investigate the relationship of organisational climate with workplace face-to-face bullying and cyberbullying. Results (*b* = −0.491, *t* = 8.4666, *p* < 0.05) indicated that organisational climate, with its dimensions, is negatively associated with face-to-face bullying. Likewise, organisational climate also shows a negative relationship with cyberbullying, as the values in the same table (*b* = −0.453, *t* = 0.059, *p* < 0.05) confirm the negative association. Hence, hypotheses 1 and 2 are accepted. At the same time, organisational climate has negative associations with emotional and physical health damage.

Research also proved that the use of technology at the workplace, including ICT and social networking, is negatively related to both face-to-face bullying (*b* = −0.310, *t* = 5.241, *p* < 0.05) and cyberbullying (*b* = −0.339, *t* = 5.563, *p* < 0.05), which leads to the acceptance of hypotheses 3 and 4. Similarly, technology usage had a positive relationship with health damage.

Face-to-face bullying was positively related to emotional health damage (*b* = 0.244, *t* = 4.002, *p* < 0.05) and physical health damage (*b* = 0.354, *t* = 4.267, *p* < 0.05). Furthermore, the relationship of cyberbullying with emotional health and physical health damage was also analysed. Results determined that the path coefficient between cyberbullying and emotional health damage was 0.104, *t*-value was measured as 1.849 and one-tail *p*-value was 0.028. Hench, Hypothesis 7 is accepted. However, cyberbullying showed no relationship with physical health damage, with *b* = −0.064 and *t* = 0.855 but *p* = 0.202; this indicates the rejection of Hypothesis 8.

Hypotheses 9–12 were related to the moderating effects of emotional intelligence on the relationship of workplace bullying (face-to-face and cyber) with health damage.

The current study results indicated that emotional intelligence moderates the relationship of face-to-face bullying with physical health damage, with *b* = −0.128, *t* = 2.229 and *p* = 0.042. Emotional intelligence also showed a moderating effect between cyberbullying and emotional health damage, with *b* = 0.104, *t* = 2.991 and *p* = 0.081. Meanwhile, no moderating effect of emotional intelligence was found on the relationship between workplace bullying and physical health damage.

## 5. Conclusions

The empirical evidence proves that organisational climate and technology usage at the workplace are precursors of face-to-face bullying and cyberbullying among Pakistan’s service sector employees. Weak leadership, poorly defined job descriptions, tough working conditions, time pressure, uncontrolled technology usage and social networking through digital technologies are the major reasons that create and promote an organisational climate that inculcates both face-to-face bullying and cyberbullying behaviour at the workplace. Abuse of ICT and digital technologies at the workplace has increased the vulnerability to cyberbullying. Technology usage at the workplace is largely attributed with shaping individual behaviour at the workplace. If it is not used appropriately, it may induce hostile actions that lead a person to becoming involved in face-to-face bullying. Research has also revealed that cyberbullying might happen at work using different technology mediums, including ICT, digital technologies and online social networking. Management should recognise that though technological tools are essential for doing business, it is equally important to effectively incorporate them into business actions and prevent them from distorting personal and work limitations.

Keeping in view that studies in the past have examined the outcomes of only one type of bullying at a time and mostly examined the effects of face-to-face bullying only, the focus of the current study was broader. It analysed the impact of both types of bullying (face-to-face and cyber) jointly on emotional and physical health outcomes. So, face-to-face bullying causes emotional and physiological distractions, but cyberbullying also has the same negative ramifications and stimulates feelings of disappointment, anger and despair and causes emotional and physiological disruptions. This study reinforced the findings of an earlier study by Katzer [73], which indicated the psychological and emotional effects of cyberbullying, like lower self-esteem, and another study, by Didde et al. [74], that concluded depression as a worker health consequence of cyberbullying.

Conversely, suppose the workers are emotionally intelligent, self-aware of their emotions, have a high degree of self-regulation, are self-motivated and are equipped with social skills and empathy. These abilities are crucial in preventing the adverse effects of face-to-face bullying and cyberbullying on employee health. Conclusively, bullying victimisation will not affect a worker’s health if the worker is emotionally intelligent.

Previous researchers have suggested that emotional intelligence enhances an individual’s skills, helping in dealing with challenging circumstances that might be damaging emotionally [55]. Likewise, Tsaousis and Nikolaou [44] also emphasised that organisations must endeavour to prevent emotional health issues among their workers, and emotional intelligence seems to reduce the possibility of emotional distress. Oginska-Bulik [75] also proposed that emotional coping might reduce occupational stress and alleviate work and organisational stress [54]. Some of the past research has also suggested that an emotionally intelligent person may have control over his/her emotions, which will induce behaviours that may help in stress reduction [76], conflict management and ethical concerns [50]. Though researchers have not studied emotional intelligence as a coping strategy to reduce the adverse health effects of workplace bullying, it is verified that emotional intelligence acts as a coping strategy to reduce the adverse health outcomes of workplace bullying, i.e., emotional and physical impact, by way of its moderating effects.

The study concluded that in the context of 500 organisations operating in four service sectors of Pakistan, workplace bullying originates from a hostile and antagonistic organisational climate, such as incompetent leadership of management, unclear job descriptions, role conflicts and high time pressure. As in the digitalisation era, technological use is also increasing day by day. Rapid technology adaptation in an organisation’s workplace also acts as a precursor/predecessor of cyberbullying and face-to-face bullying. This bullying victimisation leads workers towards emotional and physiological distractions, supported by Parkins et al. [42] and Quine [77]. Workplace bullying (either face-to-face bullying or cyberbullying) severely affects worker health and produces severe emotional and physiological health damage.

Emotional intelligence moderates the relationship between workplace face-to-face bullying and cyberbullying and worker health outcomes in terms of emotional and physiological health. Workers with strong emotional intelligence are most likely to have higher levels of self-awareness, a good sense of self-regulation, high motivation, empathetic skills and instilled social skills. These abilities are vital in coping with adverse effects of bullying victimisation in a more positive manner. So, emotional intelligence helps in mitigating the negative health effects of face-to-face bullying and cyberbullying.

### Future Agenda

Although the study provides a detailed analysis of the direct and moderating effects of variables, the cross-sectional nature of the research limits the generalisability of the conclusions, and results might be biased until or unless a longitudinal design for data collection is adopted. Though a larger sample of officials was taken for the current study, the research is limited to the headquarters/main branches of 20 service sector organisations in different cities due to time constraints. Further research may include the rest of the service sector organisations as well as the manufacturing sector. Although face-to-face bullying and cyberbullying are facets of workplace bullying, each concept is multidimensional and multifold. It is essential to investigate these concepts in a technological context to provide a broader set of rules and regulations that will help reduce such practices at the workplace.

## Figures and Tables

**Table 1:** Construct reliability, composite reliability and convergent validity.

Construct	Items	Factor Loading	Cronbach’s Alpha	Composite Reliability	Average Variance Extracted (AVE)
Organisational Climate	Job description	0.866	0.910	0.933	0.736
	Leadership	0.832			
	Time pressure	0.794			
	Cultural norms	0.862			
	Working conditions	0.930			
Technology Usage	Technology usage	0.901	0.892	0.934	0.822
	ICT usage	0.889			
	Social networking	0.930			
Face-to-Face Bullying	TB2	0.781	0.965	0.968	0.613
	TB3	0.663			
	TB4	0.724			
	TB6	0.760			
	TB7	0.757			
	TB8	0.756			
	TB9	0.765			
	TB10	0.750			
	TB11	0.793			
	TB12	0.792			
	TB13	0.797			
	TB14	0.856			
	TB15	0.805			
	TB16	0.803			
	TB17	0.796			
	TB18	0.849			
	TB19	0.703			
	TB20	0.862			
	TB21	0.830			
Cyberbullying	CB1	0.850	0.954	0.960	0.668
	CB10	0.796			
	CB12	0.859			
	CB13	0.613			
	CB2	0.779			
	CB3	0.786			
	CB4	0.821			
	CB5	0.856			
	CB6	0.826			
	CB7	0.850			
	CB8	0.867			
	CB9	0.868			
Emotional Health Damage	BOD	0.899	0.847	0.908	0.767
	BOEE	0.885			
	BOR	0.842			
Physical Health Damage	PHY2	0.710	0.926	0.937	0.554
	PHY3	0.726			
	PHY4	0.651			
	PHY5	0.691			
	PHY6	0.708			
	PSYH1	0.740			
	PSYH4	0.652			
	PSYH5	0.814			
	PSYH6	0.771			
	PSYH9	0.803			
	PSYH10	0.838			
	PSYH11	0.796			
Emotional Intelligence (EI)	EI1	0.727	0.943	0.950	0.501
	EI2	0.714			
	EI4	0.640			
	EI5	0.706			
	EI6	0.778			
	EI7	0.710			
	EI8	0.698			
	EI9	0.805			
	EI10	0.671			
	EI11	0.680			
	EI13	0.796			
	EI14	0.720			
	EI15	0.799			
	EI16	0.820			
	EI18	0.704			
	EI20	0.656			
	EI21	0.645			
	EI26	0.782			

ICT = Information and Communication Technologies, TB = traditional bullying (face-to-face bullying), CB = Cyber bullying, BOD = Depersonalization, BOEE = Emotional Exhaustion, BOR Reduced personal accomplishment, PHY = Physical health damage, EI = emotional Intellegence.

**Table 2:** Fornell–Larcker criterion.

Construct	CB	EH	EI	OC	PH	TU	F2FB
Cyberbullying	0.817 *						
Emotional Health	0.730	0.876 *					
Emotional Intelligence	−0.590	−0.695	0.708 *				
Organisational Climate	−0.735	−0.757	0.600	0.858 *			
Physical Health	0.697	0.615	−0.698	−0.731	0.744 *		
Technology Usage	−0.716	−0.641	0.571	0.632	−0.665	0.907 *	
Face-to-Face Bullying	0.602	0.738	−0.559	−0.749	0.730	−0.719	0.783 *

* indicates Square root of AVE.CB, cyberbullying; EH, emotional health; EI, emotional intelligence; OC, organisational climate; PH, physical health; TU, technology usage; F2FB = face-to-face bullying.

**Table 3:** Heterotrait-monotrait (HTMT) ratio.

Construct	CB	EH	EI	OC	PH	TU
Emotional Health	0.805					
Emotional Intelligence	0.605	0.757				
Organisational Climate	0.784	0.761	0.631			
Physical Health	0.727	0.713	0.724	0.787		
Technology Usage	0.775	0.733	0.605	0.713	0.716	
Face-to-Face Bullying	0.736	0.811	0.563	0.796	0.768	0.771

CB, cyberbullying; EH, emotional health; EI, emotional intelligence; OC, organisational climate; PH, physical health; TU, technology usage; F2FB, face-to-face bullying.

**Table 4:** The f^2^ values.

Path	Effect Size	Effect Level
Organisational Climate -> Face-to-Face Bullying	0.182	Moderate
Organisational Climate-> Cyberbullying	0.149	Moderate
Organisational Climate-> Emotional Health Damage	0.153	Moderate
Organisational Climate-> Physical Health Damage	0.065	Small
Technology Usage-> Face-to-Face Bullying	0.072	Small
Technology Usage-> Cyberbullying	0.083	Small
Technology Usage-> Emotional Health Damage	0.022	Small
Technology Usage-> Physical Health Damage	0.020	Small
Face-to-Face Bullying-> Emotional Health Damage	0.035	Small
Face-to-Face Bullying-> Physical Health Damage	0.068	Small
Cyberbullying -> Emotional Health Damage	0.060	Small
Cyberbullying -> Physical Health Damage	0.002	Small
Emotional Intelligence-> Emotional Health Damage	0.217	Moderate
Emotional Intelligence-> Physical Health Damage	0.239	Moderate

**Table 5:** Goodness-of-fit (GOF) index.

Construct	AVE	R^2^
Organisational Climate	0.736	
Technology Usage	0.822	
Face-to-Face Bullying	0.613	0.59
Cyberbullying	0.668	0.58
Emotional Health	0.767	0.71
Physical Health	0.554	0.69
Emotional Intelligence (EI)	0.501	
Average Communalities (AVE)	0.665	
Average R^2^	0.643	
AVE × R^2^	0.43	

**Table 6:** Fit summary.

Criterion	Saturated Model	Estimated Model
Standardised root-mean-square residual (SRMR)	0.043	0.062
d_ULS	1.231	2.358
d_G	0.850	1.395
Chi-square	26220.323	26545.890
Normed fit index (NFI)	0.901	0.892

**Table 7:** Hypothesis testing results.

Path	Path Coefficient	Standard Deviation	T-Statistics	*p*-Value
Organisational Climate -> Cyberbullying	−0.453	0.059	7.654	0.000
Organisational Climate -> Face-to-Face Bullying	−0.491	0.058	8.466	0.000
Organisational Climate -> Emotional Ill-Health	−0.419	0.058	7.314	0.000
Organisational Climate -> Physical Health Issues	−0.285	0.058	4.922	0.000
Technology Usage -> Cyberbullying	−0.339	0.061	5.563	0.000
Technology Usage -> Face-to-Face Bullying	−0.310	0.059	5.241	0.000
Technology Usage -> Emotional Ill-Health	0.151	0.069	2.259	0.014
Technology Usage -> Physical Health Issues	−0.005	0.065	0.083	0.934
Face-to-Face Bullying -> Emotional Ill-Health	0.244	0.062	4.002	0.000
Face-to-Face Bullying -> Physical Health Issues	0.354	0.083	4.267	0.000
Cyberbullying -> Emotional Ill-Health	0.104	0.061	1.849	0.028
Cyberbullying -> Physical Health Issues	−0.064	0.081	0.855	0.202
Emotional Intelligence (EI) -> Emotional Ill-Health	−0.322	0.043	7.469	0.000
Emotional Intelligence (EI) -> Physical Health Issues	−0.350	0.045	7.799	0.000
Moderating Effect 1 -> TB-Physical Health Issues	−0.161	0.046	1.103	0.040
Moderating Effect 2 -> TB-Emotional Ill-Health	−0.128	0.042	2.229	0.027
Moderating Effect 3 -> CB-Emotional Ill-Health	0.104	0.081	2.991	0.077
Moderating Effect 4 -> CB-Physical Health Issues	0.126	0.052	0.557	0.114

## Data Availability

Data availability is subject to the approval from participants of the survey. During the survey it was promised that data will be kept confidential and will be used for this project only.
